# Mentorship is not co-authorship: a revisit to mentorship

**DOI:** 10.1186/s13059-020-02226-6

**Published:** 2021-01-04

**Authors:** Lana X. Garmire

**Affiliations:** Department of Computational Medicine and Bioinformatics, University of Michigan, Ann Arbor, MI USA

A recent paper published in *Nature Communications* stated that “increasing the proportion of female mentors is associated not only with a reduction in post-mentorship impact of female protégés, but also a reduction in the gain of female mentors.” This paper has provoked a wide range of criticism from the scientific community for its sexism that undermines the role of female mentors, with numerous tweets and open letters to *Nature Communications*. While the outcome of this paper is yet to be determined [1], one clear flaw among others in this paper is to conflate “co-authorship with more senior women” with “informal mentorship.” It is therefore compelling to revisit the very concept of mentorship in academia for the purpose of improving diversity and gender equality.

A mentorship is a relationship between two people where the individual with more experience or knowledge is committed to pass along what they have learned to a more junior individual. The more senior individual acts as the mentor, and the more junior individual is the mentee. In academia, mentor and mentee together are engaged in many activities, such as helping the mentees to advance in their academic programs, imparting intellectual knowledge, giving advices on career navigation, and assisting the mentees through difficulties in their academic pursuit. Co-authorship may be one of the activities they engage together. It is important to note that co-authorship is not, and should not be, a necessary condition to establish mentorship, especially not for “informal mentorship.”

Good mentorship is critical for success. Almost every successful academic has benefited from mentorship along their professional journey. It is so important that NIH has set up specific funding mechanisms to foster such relationships in K-series awards. I benefited tremendously from such a mechanism to launch my own career, and I am now mentoring other junior scientists. What should one look for in a mentor? How do you know someone would be a good mentor to you? In general, a good mentor should have these following characteristics:

The mentor has similar training or educational background. It is important that you and your mentor share some “common language” in the research field. For junior faculty in bioinformatics, some faculty in biomedical informatics-related domains, familiar with publishing, grantsmanship, and institutional environment, are good fit. For postdoctoral fellows, a good mentor candidate is someone who understands your research projects and can give good suggestions or feedback as well as career guides. For graduate students, the mentorship may involve more hands-on training from someone who is patient and experienced at troubleshooting.

The mentor has a genuine interest to help, making themselves available for you. The mentor needs to have genuine interest to foster your growth professionally (rather than his/her own gains) and have time for you. It is better to ask question related to availability up front, rather than finding out later that they have too many other obligations. For example, you can ask what are the expected frequencies to have mentor-mentee meetings. if they can respond to you emails within normal business days. and how much heads-up time do they need for helping out on paper or grant submissions. If you do not feel comfortable to ask the mentor directly, consult the other lab members to find the answers.

The mentor has friendly personality that you feel comfortable to interact with. Each mentor has his or her unique style to manage and lead the group. The first impression tells a lot about the mentor’s personalities. Does he/she listen to you? Does he/she get excited about your thoughts and ideas? Does he/she give you suggestions and resources and follow up with you? Is he/she supportive and yet challenge you to do better in research? When different perceptions arise, you should feel comfortable to discuss the differences and understand each other’s viewpoints, identify a solution, and work past the issues.

The mentor has experience to help you through the problems. For junior faculty in bioinformatics, some faculty in biomedical informatics-related domains, familiar with publishing, grantsmanship, and institutional environment, are good fit. For postdoctoral fellow, someone who understands your research projects and can give good suggestions or feedback as well as guiding career development is a good mentor candidate. For graduate student, the mentorship may involve more hands-on training from someone who is patient and experienced at troubleshooting, besides the expertise and knowledge.

The mentor is trustworthy. A mentor is someone who has your back, whom you can speak honestly without worrying about confidentiality. To avoid conflict of interest, it may be good for junior faculty to find a mentor in another academic unit or even another institute. For students and postdoc fellows, the role of the primary mentor is even more critical for success. Before committing to a mentor-mentee relationship, it is a good idea to do your homework and find out their reputations among past and current colleagues, as well as past and current mentees.

Each mentor has their approaches and perceptions towards mentorship, influenced by their own trainings. When possible, it is a good idea to have a committee of mentors or co-mentors composed of both male and females, to avoid potential “biases.” Female mentors play irreplaceable and essential roles to ensure the full development of mentees. Several studies have revealed the disparities that hit female scientists particularly hard due to the on-going COVID pandemic [2, 3]. Many female mentors are overly burdened with childcare or other dependent care responsibilities besides research and mentorship, in COVID time. It is therefore very important for both mentors and mentees to understand and empathize each other’s situations, be more flexible, and make necessary adjustments.

A few reports have suggested some tips for female investigators [4, 5], which mentees may also benefit from. Due to the situation of working from home, mutual trust and respect are paramount for such a relationship. Regular communications, or even just brief check-in between mentors and mentees, are keys for keeping the connections, both professionally and emotionally. Mentor and mentee need to set up realistic goals and expectations for each other and prioritize mental and physical health and well-being over research productivities. It should be perfectly acceptable for both mentees and mentor to have online meetings with their children’s presence, when no other childcare options are available. There should be plenty of flexibility in working hours. In this long, mentally, and emotionally draining pandemic, teamwork, collaboration, and solidarity are even more essential to help the team to pull through. The pandemic will be over 1 day; a good mentor-mentee relationship is however long lasting.

## References

[1] AlShebli B, Makovi K, Rahwan T (2020). The association between early career informal mentorship in academic collaborations and junior author performance. Nat Commun.

[2] Gabster BP, van Daalen K, Dhatt R, Barry M (2020). Challenges for the female academic during the COVID-19 pandemic. Lancet.

[3] Vincent-Lamarre P, Sugimoto C, Larivière V. The decline of women’s research production during the coronavirus pandemic. Nat Index. 2020;19. https://www.natureindex.com/news-blog/declinewomen-scientist-research-publishing-production-coronavirus-pandemic.

[4] Kreeger PK, Brock A, Gibbs HC (2020). Ten simple rules for women principal investigators during a pandemic. PLoS Comput Biol.

[5] Garmire LX. From Hawaii to PECASE award: tips of success from a female bioinformatician. Genome Biol. 2019; 20(1):271. 10.1186/s13059-019-1886-x. 10.1186/s13059-019-1886-x PMC6907218 31831020

